# Challenges of achieving sustainable community health services for community case management of malaria

**DOI:** 10.1186/s12889-018-6040-2

**Published:** 2018-10-01

**Authors:** Michelle D. S. Boakye, Collins J. Owek, Elizabeth Oluoch, Juddy Wachira, Yaw A. Afrane

**Affiliations:** 1 Department of Nursing, School of Medicine and Health Science, Central University, Tema, Ghana; 2 Kenya Red Cross Society, Global Fund Programme Management Unit, Nairobi, Kenya; 3 Mild May International, Kisumu, Kenya; 4 Department of Behavioral Sciences, School of Medicine, Moi University, Eldoret, Kenya; 5 Department of Medical Microbiology, College of Health Sciences, University of Ghana, Accra, Ghana

**Keywords:** Community case Management of Malaria, Challenges, Sustainability, Community health workers

## Abstract

**Background:**

Community Case Management of malaria (CCMm) using Community Health Workers (CHWs) is an approach to improve access to timely and effective malaria case management in malaria endemic countries. So far the programme has been shown to be effective in many communities in sub-Saharan Africa. However, questions remain on the sustainability of this programme due to the high dropout cases of CHWs given their modest remuneration. The aim of the study was to identify challenges of achieving sustainable community health services for CCMm.

**Methods:**

A community based qualitative study was conducted in five districts in western Kenya where CCMm was being undertaken. In-depth interviews and focus group discussions were conducted with the CHWs, mothers of children under-five years and key informants such as public health officers and clinicians involved in the CCMm. The interviews were audio recorded and conducted in English, Swahili and the local language. Recorded interviews were transcribed. Analysis was conducted using NVivo version 7 software, where transcripts were coded after which themes related to the objectives of the study were identified.

**Results:**

The community members, the CHWs and stakeholders perceived CCMm as an important approach for reducing the burden of malaria. Key informants perceived lack of basic supplies (RDTs, gloves), drugs, inadequate remuneration of CHWs and lack of basic working equipment as challenges for CCM. CHWs highlighted that lack of drugs and basic supplies such as gloves at the health facilities, inadequate community sensitization by health workers, inadequate stipend to meet basic needs, as challenges of achieving sustainable CCMm. Some clinicians perceived that CHWs should not be given Artemisinin-based combination therapy (ACT) as part of the CCMm since they might misuse them.

**Conclusion:**

This study shows that for CCMm to be sustainable, concerted efforts from stakeholders are needed to boost the programme. Commodities needed for implementation of the programme need to be readily available and the morale of the CHWs who undertake CCMm needs boosting.

## Background

With an estimated 214 million cases of malaria worldwide with 429,000 deaths in 2015, malaria continues to burden the lives of several people especially that of Africans. Approximately 90% of all malaria deaths in 2015 occurred in Africa with about 292 000 African children dying before their fifth birthday because of malaria [1]. Many sick children in developing countries die at home because they do not have access to health facilities [2]. Following the WHO recommendations, most African countries scaled up Community Case Management of Malaria (CCMm) as an integral part of their health care delivery system, enhancing community access to health care by providing services at household and community levels. As a result, malaria burdened countries such as Kenya adopted CCMm as an approach to improve access to prompt and effective malaria case management in communities [3]. CCMm is based on the WHO recommendation that well-trained and supervised community health workers (CHWs) can provide prompt and adequate treatment to fever cases within 24 h to help reduce morbidity and mortalities due to malaria among under-five children in Africa [4].

The goal of CHWs in CCMm is to provide treatment in uncomplicated malaria and promptly refer patients beyond their level of training to the nearest health facility for further treatment [5]. With CCMm, the CHWs diagnose malaria with rapid diagnostic tests (RDTs) and treat with Artemisinin-based combination therapy (ACT) to provide prompt and adequate treatment for fever cases within 24 h to help reduce morbidity and mortality associated with malaria among under-five children in sub-Saharan Africa [1]. The services that CHWs provide promote equitable expansion of coverage for a range of preventive, promotive and curative health services [6, 7].

CCMm delivered by CHWs has proven to be an effective approach for malaria treatment in several countries [8–11] as it comes with free drugs, no transportation for treatment, availability of a care-provider even when health facilities are closed and also frequent follow-up by the CHWs for the community members. In spite of these benefits to the community, the CCMm programme faces implementation challenges that could undermine its sustainability. We therefore, conducted a qualitative study to explore the challenges that influence the sustainability of CCMm.

## Methods

### Study sites

The study was conducted in five districts in western Kenya where CHWs were undertaking CCMm. The study sites and their characteristics are described in a previous manuscript [12]. These districts were Kisumu West, Nyando and Muhoroni which were in lowland hyperendemic region and, Kisii and Kenyenya which are in the epidemic prone highlands of western Kenya.

### Study participants and sample size

The study participants, the selection criteria and the number of participants interviewed are described in full in our previous publication [12]. Briefly, we interviewed pregnant women and mothers/guardians of under-five children who had received CCMm services from the CHWs. We also interviewed the CHWs themselves and key informants that included Community Health Extension Workers (CHEWs) who supervised the CHWs, clinicians and heads of health facilities in which the CHWs are attached to, Public Health Officers (PHO) and County and District and Medical Officers of Health.

Two community units were selected in each of the five districts for the study. In each community unit, ten (10) mothers/guardians of children under the age of five years who have enjoyed the services of the CHWs, were selected for interviews through focus group discussion. Therefore there were 100 community members in total that were selected for the study from the 10 community units within the 5 districts. From each community unit, 10 CHWs were randomly selected for interviews; making a total of 100 CHWs from 10 community units in five districts. Again, in each of the community unit, the CHEW who supervises the CHWs was picked for interview; therefore 10 CHEWs were selected for interviews in the 10 community units in the five districts as key informants. The clinician and heads of health facilities in each community unit where the CHWs are attached were also interviewed. Therefore, all together, 10 clinicians who were heads of institution were interviewed as key informants. In each of the five districts we interviewed the Public Health Officer (PHO) and the district or county director of health [12].

### Study design and sampling technique

A qualitative cross sectional study was undertaken in which in-depth interviews and focus group discussions (FGDs) were undertaken with the study participants using semi-structured and structured interview guides. Two community units where CCMm were being undertaken by CHWs were picked from each district by convenient sampling.

As described in our previous manuscript, [12] all CHWs and pregnant mothers and/or caregivers of under five children from households within the same community unit (CU) and who have ever been served by a CHW were brought together at the health facility to be interviewed through FGDs. Key informants that included the CHEWs, heads and clinicians of health facilities, Public Health Officers, and County and District Medical Officers of Health were interviewed separately in their offices.

### Data collection

Interview guides were developed and were piloted in a non-study community in the Kisumu West district of western Kenya. The FGDs and key informant interview guide included questions that related to the views of the various categories of people interviewed on the sustainability of the CCMm programme. The questions were varied based on the category of person being interviewed so as to capture different themes and to answer different questions. The interview guides were first written in English, translated into the local language (Dholuo or Swahili) and later back translated into English by a different person to ensure accuracy. The data collected were qualitative and were recorded by two audio recorders. FGDs for CHWs and mothers from the communities were rendered in the local language (Dholuo and Kiswahili which are widely spoken and understood) and were later translated into English. The key informants were interviewed in English.

All study participants signed a consent form after they were adequately informed of the purpose of the study. The study was undertaken from May to September 2014.

### Data management and analysis

The data were recorded by two audio recorders and were later transcribed, translated, coded and organized into themes based on the responses of the interviewees. All the transcripts were read as individual wholes to gain an overall understanding of the data. Transcripts were coded using NVivo version 7 software. A common induction approach to coding based on phenomenology allowed us to explore the perception of the different categories of people on the challenges of achieving sustainable CCMm [13]. The results of our coding was compared for consistency of text segmentation and code application. When results were acceptable and consistent, the coding continued with periodic checks for continued intercoder agreement.

## Results

### Demographic characteristics of respondents

The median age of mothers interviewed in the FGDs was 24 years (range 19–36 years). The median age of CHWs was 32 years (range 25–59 years) CHEWs were aged between 32 and 47 years with median age of 37, whilst the ages of the clinicians and district/county director of health ranged from 28 to 52 years. Among the clinicians and PHOs that were interviewed, 40% (8/20) of them were female. Majority of the CHWs (64%) have had their education up to secondary school with the rest having finished primary education whilst only 22% of the mothers had finished secondary school, with 54% finishing primary education and 24% with no education. None of the participants declined to be part of the study [12].

### General comments

In general, all participants felt that CCMm being undertaken by the CHWs was crucial in the efforts to mitigate the spread and effects of malaria. They perceived the benefits of CCMm as: i) Providing prompt access to and timely initiation of treatment for community members and their children infected by malaria ii) availability of free malaria treatment at the comfort of their homes, iii) avoiding walking long distances from their homes to the health facilities, iv) avoiding long queues at the health facilities among others.

Despite these benefits the CCMm programme has several challenges that needs to be overcome to make it sustainable. These include the fact that clinicians at the hospital thinks that CHWs might over treat clients in the community leading to drug resistance, the lack of a constant remuneration for the CHWs undertaking CCMm, the erratic supply of drugs, basic consumables and accoutrements needed by the CHWs to perform their duties. These are discussed below:

### Perception of key informants (CHEWs, health facility in-charges and PHOs) on CHWs challenges to undertake CCMm

#### Clinicians view on CHWs dispensing drugs in the community

A number of clinicians were of the view that CHWs should not be given drugs to use in the community as part of the CCMm. They perceived that the CHWs would misuse of the drugs by giving the wrong dosage. Overdose of drugs to anyone with malaria, they said, will lead to adverse effects while over prescription could also lead to drug resistance. Children and infants are given ACTs based on their weights and CHWs who are not well vexed in these calculations might give the wrong doses. Again, their assertion is that sometimes the person might not be suffering from malaria alone but other ailments which the CHWs might not be able to diagnose or even treat.

The clinicians are of the view, that CHWs could do the diagnosis of malaria in the community with the RDTs, then give some pain killers to anyone who might have fever and encourage the patient to go to the nearest health facility with a referral note. They should not commence anti-malaria treatment but rather the patient has to be observed by the clinician in the health facility before drugs are prescribed.

Because of this reason many clinicians who are in charge of health facilities have not been giving anti-malaria drugs to the CHWs but only give out pain killers and RDTs for their work in the communities.

#### Lack of drugs and basic supplies

According to the CHEWs and PHOs, sometimes anti-malaria drugs and also supplies such as the RDTs are occasionally out-of-stock in the health facilities. CHWs could go for long periods without RDTs, gloves and other supplies they need for their work. This is quite frequent making their work difficult. This means that the CHWS sometimes do not have access to commodities that they need to work with in the communities. This is challenging for the CHWs since they cannot get drugs for their clients. Also, clients that they refer do not get drugs at the health facilities. Some community members therefore do not appreciate the CHWs referring them to the health facilities.

*“.......the drug cannot be obtained from the hospital and the hospital go for long periods without the drugs.”* PHO in a KII.

*“One area that needs improvement is the area of procurement of commodities, drugs and the gloves for the CHW, because for now there is still no clear channel for the CHWs. There comes at times that they can even go up to a whole month even without RDTs and drugs.”* CHEW in a KII.

#### Remuneration of CHWs

CHWs are the main pillars behind the CCMm. Their motivation is crucial in achieving sustainability with the programme. The key informants think that for CCMm to be successful, CHWs should be well motivated which include they need to be compensated well for their time and not just be considered volunteers. Volunteerism they claim cannot be sustained for many years. There was a feeling among the CHEWs and PHOs that the stipend that are paid to the CHWs cannot meet the basic needs of the CHWs. The CHWs are primarily volunteers from the community however, they get a monthly stipend of KES 2000 ($20) from NGOs working in partnership with the Ministry of Health that are stakeholders in malaria control. Whatever they get also experiences long delays.

*“........ I think that the salary they are getting is not enough and worse off, it sometimes delays for several months before they get it.”* PHO in a KII.

#### Lack of basic working equipment

The key informants think that one of the ways that could sustain the CCMm programme is to make sure that CHWs are well equipped for the task of undertaking the CCMm in the community. CHEWs think that the CHWs are challenged on a number of working gears, which include basic supplies like raincoats or umbrellas and gumboots they need to perform their functions when it is rainy or when it is muddy. They also think that since sometimes CHWs are called at night to attend to clients, their security could be enhanced by being given torchlights otherwise they could be robbed or even bitten by a snake on their way to a clients place. The clinicians assert that the CHWs do not have any means to reach their clients’ houses but just walk to the place by themselves. They think that CHWs will benefit greatly and their output enhanced if bicycles are given to them to perform their functions instead of walking from one compound to another.

#### Lack of trust by some community members

Some community members do not trust the CHWs for the reason that they are not adequately trained to handle some health services. Though these are few people who have this belief, the feeling was that they might infect more people with their ideas.

### Perception of CHWs on challenges of CCMm

#### Conflicting information from healthcare workers

The CHWs asserts that a lack of clear information about health facility charges to clients by some health care workers makes it difficult for them to be trusted by some community members, particularly on malaria treatment that is considered free of charge. Malaria diagnosis and treatment are supposed to be free, according to the government policy, but in practice this is not so with the health facilities who charge fees for both because they claim the government does not send them any money to cover free drugs and supplies for diagnosis. Where the drugs are supplied free of charge by the government, they are most of the time out of stock in the health facilities. These are challenges to the sustainability of CCMm.

#### Lack of drugs at the health facilities

Some of the health facilities that the CHWs refer their clients to lacked anti-malarial drugs some of the times. The CHWs indicated that lack of drugs in health facilities leads to lack of trust by community towards their services. They assert that, they are not given drugs by the health facilities to give to the clients and when they refer clients to the health facilities too, there are no drugs available to give to clients.

Also, the malaria test at the laboratory of the health facility is not free unlike the RDT test of the CHWs in the community which the CHWs do not charge.

*“...when you give someone a referral, they think that they will also be given the medicine but when they get to the hospital and find none, or they are told to buy on their own they come back thinking you are the bad guy.”* CHW from FGD in Kisii.

*“.....at times you give someone a referral note, they go to the hospital thinking that they will be served for free, but they get there and are tested with their own money and when they are found positive, they are told again to go to the chemist and buy the medicine. So they find it useless because they were not given any medicine.”* CHW from FGD in Kisii.

#### Lack of basic supplies and drugs at the disposal of the CHWs

CHWs are challenged by the lack of basic supplies and drugs to support their clients with. Particularly gloves, malaria testing kits, bed nets and painkillers.

*“Unfortunately they gave us the RDTs and little drugs but no pain killers and gloves, and we are buying them for our clients.”* CHW from FGD in Enderege, Kisii County.

The CHWs also claim that sometimes a pack of RDT is shared between 2 or more CHWs. Each pack also has one buffer solution that is supposed to run the test. Therefore sometimes they do have the RDTs but no buffer because the buffer solution might be with another CHW who might be using it in a distant community.

#### Logistical support for the work of CHWs

CHWs reported a lack of the proper logistical support to undertake their work. These include provision of essential protective clothing such as gumboots, umbrellas or raincoats to walk through the rain to see their clients.

The CHWs are most times challenged to travel far distances to attend to some of their clients. Occasionally they are forced to use their own money to hire bicycle services that comes in the form of bicycle or motor bike “taxis”.

*“....something like this bicycles, the homesteads are so far, so when you receive a call it will force you to walk if you do not have money and if you have money you will use a “boda-boda” (bicycle taxi)”* CHW from an FGD in Chemelil, Kisumu County.

*“There are times that it is raining and it is at night and you have gone to a given place, you have to stand there for a long times since you do not have an umbrella and since you do not have gumboots it is hard.”* CHW from FGD in Kenyenya, Kisii County.

#### Challenges of female CHWs

Female CHWs also find it challenging attending to clients at night due to security risks of a lady walking alone at night. Younger ladies were of the view that sometimes some men pretend they are sick and call them to come to their house. They are of the view that they face more harzards than their male counterparts. But they reported that in some areas of health such as reproductive health, some ladies are more comfortable talking to them.

#### Inadequate community sensitization by health workers

Community members were not sensitized by the health workers on role of the CHWs on testing for malaria in the community, hence they faced difficulties at the initial stages.

*“...we were given these RDTs and it was not announced in the community and so it was upon us to let them know that they we have the kits and so they need to be tested before they go to the clinic.”* CHW from FGD in Kenyenya, Kisii County.

#### Inadequate stipend to meet basic needs

The CHWs find the stipend that they earn to be too little to sustain their basic needs. They are also weary of the fact that this “small money” experiences delays before they could receive it.

*“....even that 2000 is little money since it can nowadays be compared with the money that people who go for casual work can get in six days and therefore if they can remunerate us better they should think about it.”* CHW from an FGD in Ojolla A, Kisumu County.

*“....the ‘soap’(stipend) should also not come so late and we will be able to work well, like me I have four villages it is a hard work and so if this stipend is maintained at least you have a reason to work.”* CHW from FGD in Kisii.

### Perception of the women/communities on CHWs challenges

#### Lack of basic supplies and drugs

Women who have used the services of CHWs in the recent past cited the challenges faced by the CCMm as the lack of basic supplies needed by the CHWs to undertake their work such as malaria testing kits (RDT), anti-malaria drugs, pain killers, gloves. This is even more critical in some communities that public health facilities do not operate on weekends.

“ *Their work is good and they need to be added drugs since at times when you go to them they tell you that they do not have the drug and sometimes it is even on a Saturday and so for you to wait till Monday it will be hard.”* Expectant mother from an FGD in Osiri, Kisumu east district.

#### Suspicion by some community members of the services of CHWs

The women from the communities felt that some CHWs do not get support from all members of the community due to suspicion. Some of the mothers asserted that if the CHW is coming from a rival family then there is always suspicion that the CHW is coming to look for information. Others also think that CHWs could be up to mischief since they also distribute free condoms in the villages on reproductive health and HIV services which not all community members subscribe to. All these affects uptake of their services. A case in point is when a woman said her mother-in-law warned her not to entertain the CHW in the household compound again because the mother-in-law felt the CHW would bring family planning pills to the woman. She said this was based on suspicion.

#### Unfavourable weather conditions

The women felt that sometimes it becomes difficult for the CHWs to access their clients especially during rainy seasons whilst the CHWs lack the necessary equipment like umbrellas, raincoat or boots to walk in the rain.

*“.....you will call her but she will not come since she may be coming from far and because you also do not want to walk in the rain to her place you will just suffer.”* Expectant mother from an FGD in Kisumu West.

## Discussion

This study assessed the challenges in achieving a sustainable CCMm in malaria-endemic districts in Kenya. CCMm being undertaken by CHWs was found to be acceptable to the community members who showed a good attitude to it although they initially did not patronize the CCMm. They later identified and appreciated CCMm as a health service that brings health care delivery closer to their doorstep and again helped them to avoid long queues in the traditional health care settings. Community members equally liked the idea that diagnosis and treatment of malaria by the CHWs were free under CCMm. The acceptability of CHWs testing malaria using RDTs and treating uncomplicated malaria with ACT is consistent with previous studies [14–19].

It is interesting to know that community members preferred to be attended to by CHW than to go to a health facility to see a clinician when not well. This may be due to the long queues, unnecessary delays, lack of adequate attention and misconduct of some clinicians that are encountered at health facilities. They would rather see someone within their village who will test and treat them as quickly as they can so they go back to their homes.

The present study showed that CHWs have shown a commitment to undertake CCMm and to do it correctly. Other studies from other countries have also shown that CHWs were able to correctly perform RDTs, and showed high adherence to test results [5, 20]. These prove that with proper training and supervision, CHWs could be trusted to undertake CCMm in communities in sub-Saharan Africa [21]. The fact that in different countries CHWs have also been accepted by community members to undertake CCMm [22] shows that the programme can be successful if implemented well. The benefits of CCMm include prompt access to treatment to especially under five children who bear the greatest burden of the malaria disease.

For the CCMm programme to be sustainable, the CHWs will have to be more motivated well since they are the cornerstone for the implementation. Commodities such as the RDTs and drugs needs to be available in the health facilities so that CHWs can access them for their community work. Health facilities in many sub-Saharan African countries including Kenya, do experience stock-out of commodities some of the time [23] and if this gap could be bridged then it will also trickle down to the CCMm programme to make it a success.

The programme cannot be successful if clinicians, health facility-in-charges, and technicians are not part of the implementation. There seems to be a disconnect between these categories of people in the health facilities and the CHWs who are in the communities. The fact that some of the clinicians and technicians in the health facilities shared the opinion that CHWs are not adequately trained to handle patients diagnosis and treatment in the community and are not supporting them tells the fact that they might not be well informed enough of the training of the CHWs and goal of the CCMm programme. However, if healthcare workers are part of the training of CHWs and the CHWs constantly get to be reviewed and supervised by these health care workers, they all could work in harmony to implement CCMm.

The fear of many healthcare workers needs to be addressed. For instance, the clinicians fear that CHWs will misuse drugs, over treat their clients which could lead to drug resistance. CHWs might be tempted to treat everyone who presents with fever and who may test negative for malaria with the RDTs. Malaria treatment is being undertaken with powerful expensive ACT drugs [24] and if these develop resistance in people, there are no available drugs to replace them [12]. Therefore the concerns of the clinicians could be right and more training needs to be organized for the CHWs.

## Conclusion

In conclusion, there are several challenges facing the implementation of the programme that needs to be addressed for the programme to be a success. The challenges raised here are also similar to many countries in sub-Saharan Africa. Concerted efforts from governments through the ministry of health, malaria control programmes, civil society organisations, health care workers and communities are needed to boost the programme to make it sustainable and to boost the morale of the CHWs to sustain and retain their services to undertake the programme.

## Abbreviations

ACT: Artemisinin-based combination therapy
CCMm: Community Case Management of malaria
CHEWs: Community Health Extension Workers
CHWs: Community Health Workers
CU: Community unit
FGDs: Focus group discussions
KES: Kenya Shillings
KII: Key informant interview
NGO: Non-governmental organization
PHO: Public Health Officer
RDT: Rapid diagnostic tests
WHO: World Health Organization

## References

[1] World Health Organization (WHO), (2016), Fact sheet: world malaria day, http://www.who.int/malaria/media/world-malaria-day-2016/en/. Accessed 12 Sept 2017.

[2] Schellenberg JA, Victora CG, Mushi A, De Savigny D, Schellenberg D, Mshinda H (2003). Inequities among the very poor: health care for children in rural southern Tanzania. Lancet.

[3] Salako LA, Brieger WR, Afolabi BM, Umeh B, Agomo R, Asa P (2001). Treatment of childhood fevers and other illnesses in three rural Nigerian communities. Journal of Topical Pediatrics.

[4] WHO/TDR, Geneva. WHO (2008). World malaria report.

[5] Ndiaye Y, Ndiaye JL, Cisse B, Blanas D, Bassene J, Manga IA (2013). Community case management in malaria: review and perspectives after four years of operational experience in Saraya district, south-East Senegal. Malar J.

[6] Gilmore B, McAuliffe E (2013). Effectiveness of community health workers delivering preventive interventions for maternal and child health in low- and middle-income countries: a systematic review. BMC Public Health.

[7] Glenton C, Scheel IB, Lewin S, Swingler GH (2011). Can lay health workers increase the uptake of childhood immunisation? Systematic review and typology. Tropical Med Int Health.

[8] Ajayi IO, Browne EN, Bateganya F, Yar D, Happi C, Falade CO (2008). Effectiveness of artemisinin-based combination therapy used in the context of home management of malaria: a report from three study sites in sub-Saharan Africa. Malar J.

[9] Tiono AB, Kabore Y, Traore A, Convelbo N, Pagnoni F, Sirima SB (2008). Implementation of home based management of malaria in children reduces the work load for peripheral health facilities in a rural district of Burkina Faso. Malar J.

[10] Pagnoni F (2009). Malaria treatment: no place like home. Trends Parasitol.

[11] Yeboah-Antwi Kojo, Pilingana Portipher, Macleod William B., Semrau Katherine, Siazeele Kazungu, Kalesha Penelope, Hamainza Busiku, Seidenberg Phil, Mazimba Arthur, Sabin Lora, Kamholz Karen, Thea Donald M., Hamer Davidson H. (2010). Community Case Management of Fever Due to Malaria and Pneumonia in Children Under Five in Zambia: A Cluster Randomized Controlled Trial. PLoS Medicine.

[12] Owek CJ, Oluoch E, Wachira J, Estambale B, Afrane YA (2017). Community perceptions and attitudes on malaria case management and the role of community health workers. Malar J.

[13] Corbin J, Strauss A (2008). Basics of qualitative research techniques and procedures for developing grounded theory.

[14] Thiam S, Thwing J, Diallo I, Fall FB, Diouf MB, Perry R (2012). Scale-up of home- based management of malaria based on rapid diagnostic tests and artemisinin based combination therapy in a resource-poor country: results in Senegal. Malar J.

[15] Ajayi IO, Browne EN, Garshong B, Bateganya F, Yusuf B, Agyei-Baffour P (2008). Feasibility and acceptability of artemisinin-based combination therapy for the home management of malaria in four African sites. Malar J.

[16] Elmardi KA, Malik EM, Abdelgadir T, Ali SH, Elsyed AH, Mudather MA (2009). Feasibility and acceptability of home-based management of malaria strategy adapted to Sudan’s conditions using artemisinin-based combination therapy and rapid diagnostic test. Malar J.

[17] Tine RC, Faye B, Ndour CT, Ndiaye JL, Ndiaye M, Bassene C (2011). Impact of combining intermittent preventive treatment with home management of malaria in children less than 10 years in a rural area of Senegal: a cluster randomized trial. Malar J.

[18] Chinbuah AM, Gyapong JO, Pagnoni F, Wellington EK, Gyapong M (2006). Feasibility and acceptability of the use of artemether-lumefantrine in the home management of uncomplicated malaria in children 6–59 months old in Ghana. Tropical Med Int Health.

[19] Akweongo P, Agyei-Baffour P, Sudhakar M, Simwaka BN, Konate AT, Adongo PB (2011). Feasibility and acceptability of ACT for the community case management of malaria in urban settings in five African sites. Malar J.

[20] Counihan H, Harvey SA, Sekeseke-Chinyama M, Hamainza B, Banda R, Malambo T (2012). Community health workers use malaria rapid diagnostic tests (RDTs) safely and accurately: results of a longitudinal study in Zambia. Am J Trop Med Hyg..

[21] Harvey SA, Jennings L, Chinyama M, Masaninga F, Mulholland K, Bell DR (2008). Improving community health worker use of malaria rapid diagnostic tests in Zambia: package instructions, job aid and job aid-plus-training. Malar J.

[22] Mukanga D, Tibenderana JK, Peterson S, Pariyo GW, Kiguli J, Waiswa P (2012). Access, acceptability and utilization of community health workers using diagnostics for case management of fever in Ugandan children: a cross-sectional study. Malar J.

[23] Kangwana BB, Njogu J, Wasunna B, Kedenge SV, Memusi DN, Goodman CA (2009). Malaria drug shortages in Kenya: a major failure to provide access to effective treatment. Am J Trop Med Hyg.

[24] WHO (2015). World malaria report.

